# Healthcare providers’ perceptions of changes in guidelines for care of minors with gender dysphoria in Sweden: An interview study

**DOI:** 10.1371/journal.pone.0336950

**Published:** 2025-11-19

**Authors:** Elsa Svantesson, Ida Linander

**Affiliations:** Department of Epidemiology and Global Health, Umeå University, Sweden; McMaster University, CANADA

## Abstract

**Background:**

The number of youths seeking care for gender dysphoria has increased globally over the last ten years. In December 2022, the Swedish National Board of Health and Welfare published an updated knowledge support for the care of minors (children and adolescents) with gender dysphoria. This knowledge support recommends stricter criteria for prescribing puberty blockers and cross-gender hormones to minors, and differs both from previous healthcare practices and from international guidelines.

**Aim:**

This study aimed to explore healthcare professionals’ perceptions of the updated knowledge support and its impact on healthcare practices and the care seekers (minors with gender dysphoria).

**Methods:**

This qualitative interview study included 11 participants who worked clinically with gender-affirming care of minors (both evaluation and providing medical interventions). The participants came from different regions, different evaluation teams, and different professions. Reflexive thematic analysis was used.

**Results:**

While the new knowledge support was partly intended to solve geographical differences, different evaluation teams had implemented the new guidelines differently. New criteria around childhood debut, neuropsychiatric conditions, and hormonal treatment seemed to have partly changed the conditions for the evaluation. The greatest change had been regarding access to puberty blockers and hormonal treatment. Some participants argued for a larger shift towards psychosocial support, while others highlighted the tension between care seekers’ needs and the shrinking opportunities to help based on the new guidelines. Some participants perceived that people lacking in expert knowledge had affected the content of the knowledge support.

**Conclusions:**

Differing opinions of the knowledge support were expressed by healthcare providers working with gender-affirming care for minors. Some were pleased with the new guidelines and believed the new knowledge support to be clearer than the old one. Others were more critical, stating that the knowledge support had not taken clinical experience into consideration, was too open to interpretation, and was too restrictive.

## Introduction

In December 2022, the Swedish National Board of Health and Welfare (NBHW) published a new national knowledge support regarding the treatment of children and adolescents with gender dysphoria. These guidelines were commissioned by the Swedish government, developed by around fifty endocrinologists, psychiatrists, psychologists, speech therapists, lawyers and others. The knowledge support is intended to be seen as providing recommendations for medical evaluation and treatment. It recommends that puberty blockers, gender-affirming hormonal treatment, and mastectomy should only be provided to minors as part of medical research, or in exceptional cases if the minor fulfills certain criteria (see Appendix). The previous Swedish guidelines for treatment of minors with gender dysphoria, which were published in 2015, included puberty blockers, hormonal medication, and mastectomy as possible treatments [1].

The new and more restrictive knowledge support has been argued to be appropriate due to a stated lack of evidence regarding the long-term effects of puberty blockers and gender-affirming hormonal treatment, first acknowledged in a 2022 report from the Swedish Agency for Health Technology Assessment and Assessment of Social Services [2]. However, even before this, in March 2021, a hospital in Stockholm, Department of pediatric endocrinology at Karolinska University Hospital [3] had restricted its treatment of transgender children and adolescents. Another argument for the more restrictive guidelines is the increasing number of minors seeking care for gender dysphoria, especially people assigned female at birth [4,5]. A third argument is concerned with media reports of de-transitioning among young adults [1]. However, previous studies show that regret after gender-affirming surgery is low, at around 1% [6]; moreover, only 2.2% of people who obtained a legal change of sex in Sweden between 1960 and 2010 expressed regret over their choice [7].

The World Professional Association of Transgender Health (WPATH) published its most recent guidelines regarding the treatment of gender dysphoria in 2022. These guidelines recognize the quality of evidence as a key challenge regarding the treatment of children and adolescents, but note clinical benefits when treating children with gender dysphoria within multidisciplinary teams. WPATH recommends that certain criteria should be fulfilled when giving gender-affirming treatment (see Appendix 1) [4]. Hence, there are significant differences between the Swedish national recommendations and international guidelines for the treatment of gender dysphoria in children and adolescents. The Swedish NBHW says that puberty blockers and hormonal treatment should not be given until the care seeker has reached Tanner stage 3, while WPATH says Tanner stage 2. The Swedish guidelines have guardians' consent and age limits as criteria for medical treatment, neither of these things are mentioned in the WPATH’s guidelines. To get hormonal treatment in Sweden you also have to live in line with your gender identity and suffer from puberty to get puberty blockers, things that are not mentioned in the WPATH’s criteria [1].

The Dutch Protocol, which dates from 1996, was based on years of research and has set the standards for treating minors with gender dysphoria worldwide [8]. International and national guidelines worldwide are citing this Protocol.

People suffering from gender dysphoria exhibit a higher prevalence of mental illness than the rest of the population, including the prevalence of suicide, self-harm, depression, and anxiety [4,5,9]. This level of psychopathology can partly be explained by the stigma and minority stress experienced by transgender people [10,11]. While long-term studies remain limited, a growing body of research indicates that gender-affirming care is associated with improved psychosocial functioning and mental health outcomes among youth [12–15]. Puberty blockers can improve psychosocial functioning and decrease depressive symptoms in adolescents [13–16], and better mental health has been observed among transgender adults who received puberty blockers as children [17]. However, even after gender-affirming care, transgender people still show higher rates of psychiatric morbidity and mortality compared with the entire population [18]. Neuropsychiatric disabilities such as autism and ADHD are more common among youth with gender dysphoria compared to the general population, both in Sweden and internationally [4,5].

In the past few years, gender-affirming care has been widely debated not only in Sweden but all over the world [19]. Both transgender organizations and healthcare providers have expressed criticisms of the new and more restrictive knowledge support, since it reduces the possibility of using puberty blockers [19–21]. Even before the new recommendations, transgender youth had a long wait for access to care, which was experienced as affecting their mental health and social lives [22]. The updated knowledge support might entail an even longer time for care seekers to wait before getting puberty blockers and hormonal treatment, and new challenges for the profession.

The perceptions of professionals working with the targeted patient group have been largely absent in the public debate and reports. To our knowledge, this paper is among the first to describe health care professionals’ views on changing guidelines for gender-affirming care for minors. Research and proven experience are the basis of the medical evidence on which guidelines should be based, and healthcare providers possess this clinical experience. It is therefore important to recognize their point of view. This study aimed to explore healthcare professionals’ perceptions of the updated knowledge support, as well as its impact on healthcare practices and on care seekers; that is, children and adolescents with gender dysphoria.

## Materials and methods

### Study context: Gender-affirming care in Sweden

In Sweden, the need for gender-affirming care among children and adolescents is evaluated by teams including psychiatrists, psychologists, and counselors based at psychiatric or sexological clinics. The teams clinically evaluate the gender dysphoria, and screen the patient for other psychiatric conditions if this has not already been done. Referrals for further gender-affirming treatments, for example to pediatric endocrinological clinics, are then made based on the patients’ needs [23].

### Study design

This was a qualitative interview study among healthcare professionals. The inclusion criterion was being a healthcare provider who had worked clinically with gender-affirming care of minors after December 2022, when the new knowledge support was published. No exclusion criteria were applied.

### Recruitment and participants

Participants were recruited via purposive sampling. People in Sweden working with evaluation of gender dysphoria, gender-affirming medical care (e.g., handling hormonal treatment) or with referrals to gender identity clinics were contacted via email and given information about the study. Additional participants were found via snowball recruitment. Individuals who answered the email to express interest and who fulfilled the inclusion criteria were sent an information sheet about the study. The time and place of the interview were decided by the participants. Interviews were conducted between February and December 2024 by both authors. Data collection was finalized when themes were recurring in relation to the research aim [24].

The 11 participants worked in or with four of the six Swedish gender evaluation teams, and were based in five different regions. They represented different healthcare occupations such as child psychiatrist, psychologist, counselor and child endocrinologist. They had worked within care for gender dysphoria for 3–12 years.

Of the total number of potential participants approached (n ~ 23–26), around 12–15 did not reply (of which quite many probably only worked with adults), while five responded that they either did not have time, did not wish to participate or only worked with adults.

### The interviews

A semi-structured interview guide, constructed by the authors based on previous studies of the authors and research experiences, was used, with open-ended questions followed by more focused supplementary questions. The questions were divided into three areas: 1) perceptions of how the new knowledge support impacted healthcare, 2) perceptions of how the new knowledge support impacted youth with gender dysphoria, and 3) need for change and improvements. Adjustments were made to the questions on the basis of both the participant’s answers and their profession and in subsequent interviews based on insights from earlier participants.

Ten of the interviews were held via video call and one was held face-to-face. Only the participant and interviewer were present. Each interview took between 30 and 50 minutes. Notes were taken to remember follow-up questions but were not used for analysis. The interviews were transcribed verbatim by an AI software (noScribe) and then corrected by one of the authors. The transcripts were not sent back to the participants. The interviews and the analysis were performed in parallel, to validate when themes were recurrent.

### Data analysis

The material was analyzed with reflexive thematic analysis according to Braun and Clarke [25], with an interpretative approach [24]. First, the interviews were transcribed, and the transcripts were read many times in order to become familiar with the material; notes were taken during this process. Next, the material was coded by the authors, and then the codes were divided into themes and sub-themes. Following this, the themes were reviewed to find new relations between the themes and codes, and definitions and names were given to each theme and subtheme. Finally, the findings were written.

### Ethics

The project was approved by the Swedish Ethical Review Authority (ref: 2020−00929).

All procedures performed in studies involving human participants were in accordance with the ethical standards of the institutional and/or national research committee and with the 1964 Helsinki declaration and its later amendments or comparable ethical standards. Informed (recorded) consent was obtained from all individual participants included in the study.

Participation of healthcare workers in the study could be sensitive, since this subject might be experienced as controversial and has been highly debated in the past few years. This was handled by ensuring confidentiality. Personal data were kept separately from the interview files, and we present only limited information about each participant to avoid traceability. No patients can be identified in the interview material, as only general case examples were given by the participants.

## Results

The results are presented as four themes focusing on different recurrent issues discussed by the participants, each with its own sub-themes. Table 1 gives an overview of how the sub-themes were categorized into themes.

**Table 1:** Themes and subthemes.

Themes	Geographical differences as both background and result	Navigating a complex evaluation and problematic criteria	Balancing treatment access, timing, and alternative support	Opinions and debates affecting care guidelines and care providers
Sub-themes		Changing conditions for an explorative evaluation Autism is blurring the picture Changing criteria and difficult decisions	Restrictive criteria for hormonal treatment and (missed) opportunities for alternative support Postponed or denied treatment	The power of the public debate Taxing work and a questioned profession

### Geographical differences as both background and result

A recurrent subject mentioned by the participants was differences in care practices between gender evaluation teams across the country. The teams seemed to have worked with different approaches not only before the knowledge support was published, but also after. P11 summarized the situation: “*One of the ambitions of the knowledge support is that it would create more uniformity. If I jumped forward in time, I don’t think that’s how it turned out.*” P3 reflected: *“My perception is that maybe the new… that the new knowledge support has affected the different evaluation teams a little bit differently.*” The differences both before and after the publication of the knowledge support were stated to be problematic, since they meant that the healthcare was not geographically equitable. Some wished for more similarity between the healthcare practices of the teams. P4 expressed the presence of regional differences by saying: “*I believe it is consensus that is needed.*”

The new knowledge support seemed to have affected different teams differently. Several participants felt that the knowledge support could be interpreted in more than one way, which could be the reason for the differences in its impact between the different teams and hence geographical places. Regarding this vagueness, P4 said:

*If possible, clarify the exemption criteria further, particularly regarding the demand to avoid excessive complicating factors. This aspect has been the most open to interpretation, and is likely one reason for the significant variation in healthcare practices across the country.*

Other participants pointed to the criteria of childhood debut when reflecting on what might create differences between teams; for example, P11 said: “*The criteria around this with gender incongruity in childhood, how to assess it, on what grounds. What is gender incongruence in childhood?*” As the criteria did not state any specific age or how this incongruence should be expressed, there was room for differences in the interpretation and evaluation.

Representatives of some teams thought that the increased restrictions on treatment in the new knowledge support were a positive development, for example P3 said:

So I would still say that it [new knowledge support] has had a positive impact, in that… in that you see the individual child or adolescent as a whole, where the gender incongruence or gender dysphoria is one part of them. But, there are also other aspects to the child’s or adolescent’s overall constitution.

Participants from other teams were more negative and mentioned the lack of flexibility when it comes to treatment as problematic. P2 explained that for patients who had both gender dysphoria and mental illness, and who needed gender-affirming treatment, “*it gets a bit disqualifying because the guidelines are a bit too tight there.*” P4 felt that the knowledge support’s *“focus was almost just put on tighten, tighten on all fronts/…/ I would have wished for a little more trust in the evaluation teams’ evaluations.*” P11 said that many things still worked the same as before, but that before the new guidelines they “*wouldn’t have waited for the 18th birthday*”, noting that “*the guidelines are rigid*”. These results illustrate both how different teams seems to hold quite different perspectives, and the inherent tension in prescriptive guidelines: on the one hand, they can help to reduce geographical differences, but on the other hand, they may restrict care providers to make their professional context-specific assessments. This was illustrated by P9:

*I think it’s difficult to have such clear guidelines so that you get away from [differences between teams]. And I think that you may not want to get away from having that room for action either. For professionals to actually make their judgments. And use their experience and expertise. The guidelines would risk becoming very blunt./…/ But I think we’d need to have more routines and forums for the teams to work together. To make joint judgments. In order not to drift apart [more].*

In sum, while the new knowledge support was partly intended to reduce geographical differences, it seemed that different teams had interpreted and implemented the new guidelines differently, which can contribute to geographical inequitable care.

### Navigating a complex evaluation and problematic criteria

While the aim of the evaluation was perceived to be the same as before, some new criteria and different possibilities for hormonal treatment seemed to have partly changed the conditions for the evaluation.

### Changing conditions for an explorative evaluation

Several participants thought that the evaluation process was similar to how it was before, as exemplified by P2: “*We do what we had done previously.*” The participants highlighted that the aim was to have an exploratory, individualized evaluation in which the young person could reflect on their own identity, life, and future in counseling with healthcare providers for a longer time, around 2 years. Some felt that the knowledge support had increased “*the understanding between pediatric endocrine and the evaluation teams at child psychiatry*” (P8). P3 and P6 explained that even before the new guidelines, in their clinic they had already worked in the way suggested by the new knowledge support, trying to follow the so-called Dutch Protocol (which is referred to frequently in the knowledge support). P3 stated:

*Our children’s team have since before the new guidelines seen a need to work in the way that the new guidelines prescribe and allow for/…/ From the beginning, this has been the only thing we can hold on to. So there I think that we’ve tried to, like, offer this treatment based on the conditions that the Dutch Protocol prescribes.*

Although P3 argued that her team worked in a very similar way to before, she also said: “*now when the Swedish National Board of Health points more clearly to that [the Dutch Protocol]*”, indicating that they might not have strictly followed the old guidelines.

The participants stated that the new guidelines could make it both easier and more difficult to conduct the evaluation. Now, in some cases, puberty blockers were off the table from the start, as it would not be possible for the care seeker to fulfill the criteria. Some participants explained that this situation, paradoxically, left more room for the evaluation itself: without the time-sensitive pressure to decide on blockers before puberty advanced, they could focus more on exploring overall needs. However, the stricter criteria for access to care might mean that the care seekers did not share all their thoughts, experiences and feelings:

*I think the description in the international guidelines is much more helpful. That you need to be able to explore gender identity development over time. This [the knowledge support] also leads to a difficulty or obstacle for us in working with these things. We should explore things together with the patients. But when there is such a clear box that they must fit into to be considered for treatment, then there are many who could, and are, adapting their descriptions based on the fact that they know about this. This is a well-read patient group (...) Our opportunities for free exploration are much more limited.* (P9)

Relatedly, before the new guidelines, puberty blockers were used not only as a step in the transition, but also in some cases to buy time to decrease gender dysphoria and get a more productive evaluation. P2 explained: “*We used puberty blockers as a part of the evaluation, not maybe like a part of the evaluation per se, but as a way to decrease gender dysphoria and give space and time to explore who you are.*” Now, the stress about not getting puberty blockers affected not only the patient, but also the evaluation itself. P2 continued: “*It can be hard to achieve calmness in the exploration, because there is worry and stress.*”

### Autism is blurring the picture

A central difference between the new guidelines and the international ones is the formulations around “neuropsychiatric disability”. P1 was critical towards these formulations in the knowledge support, which she referred to as saying that “*nothing should blur the evaluation*” regarding neuropsychiatric disability. P9 was also critical, saying that if the guidelines resulted in a whole patient group being excluded, this would be “*highly discriminatory*”.

Some participants believed it was hard to separate autism and gender dysphoria in the evaluation. P7 asked: “*Does it have more to do with autism symptoms, for example, than with gender dysphoria and gender incongruence?*” P2 thought that autism might in some cases be “*instead of gender dysphoria, an explanation for what you’re seeing*”. P3 said: “*It’s hard to disregard that this plays a part,*” meaning that the neuropsychiatric disability affects gender dysphoria. P5 felt that some patients did not see their autism as the source of some issues, but rather thought it was gender dysphoria: “*‘Yes but if I only got these hormones, if I could only remove my breasts, then I would have done so much in life, and I would have gone to university. I wouldn’t be home.’*”

Other participants clearly stated that having autism did not mean that a person could not get treatment. P4 noted that gender dysphoria and autism could be present at the same time, and said that it was *“unfortunate when people want to explain that one thing is because of the other. We believe that both can exist.*” P1 did not think that neuropsychiatric or intellectual disability should be an exclusion criterion, and believed that the teams were competent enough to evaluate gender dysphoria despite these diagnoses: “*If care is needed, then neuropsychiatric variation or intellectual disability diagnosis, for that matter, is not in the way, of course.*”

Assessing the young person’s maturity was both a part of the evaluation and a criterion for having treatment. The participants felt that a neuropsychiatric disability might impact maturity; for example, P7 stated: “*A healthy 16–17-year-old without diagnoses has in general, in terms of maturity, gotten further than one with autism.*” In a similar vein, P2 said:

*Patients who have several diagnoses like intellectual disability and autism, but who have been clearly gender dysphoric since they were little, then the gender dysphoria is nothing hard [to diagnose], but sometimes I can think that it is not right maturity-wise to give treatment before age 18.*

In a similar way, P9 felt that neuropsychiatric diagnoses could prolong the evaluation in some cases, but in other cases “*it does not blur the picture*”. Hence, some participants pointed to the importance of individualized evaluations and assessments, which the strictness and rigidity of the new guidelines might make more difficult.

### Changing criteria and difficult decisions

Childhood debut of gender dysphoria, which is a criterion for access to medical care in the new guidelines, was another aspect of the evaluation that our participants mentioned as being difficult. As P9 said, not all young people might have the “*possibilities, preconditions, the language to understand what it has been about, or to have felt safe enough to talk about it.*” The criteria around this are now stricter than in the old guidelines, and the requirement to document the childhood debut affected the content of the evaluation. Some meant that this might favor “*stereotypical gendered expectations and norms*” (P9), meaning that such issues might be the only way to assess this childhood debut among children who had not expressed it verbally.

Even though the knowledge support gave criteria for providing treatment, a few participants thought it was hard to decide who should get gender-affirming treatment; they expressed the view that gender dysphoria was subjective, and so no test could help in the decision-making. P6 said: “*But that’s probably the hardest thing, how are we supposed to know? How are we ever supposed to know what’s right?*” Similarly, P2 said: *“Sometimes it can be easy to give a diagnosis of gender dysphoria, but the treatment decision can be something totally different.*” This was still mentioned as an issue, despite the new knowledge support.

### Balancing treatment access, timing, and alternative support

Access to puberty blockers and hormonal treatment for the care seekers seemed to be what had changed the most with the new knowledge support. Some participants argued for a larger shift towards psychosocial and alternative support, while others highlighted the tension between the care seekers’ needs and the opportunities to help within the new guidelines.

### Restrictive criteria for hormonal treatment and (missed) opportunities for alternative support

According to the participants, it was the use of puberty blockers and hormonal treatment that has changed the most for the teams, although some said that their teams had already been more restrictive even before the new knowledge support. These treatments could still be given in exceptional cases, but it was now rarer because the criteria in the new guidelines were stricter. Medical interventions were “*simply becoming less relevant in the children’s team*” (P5). However, some said that even before the new guidelines, medical care was provided only in “*exceptional cases*” (P9).

The timing and age limits in the new guidelines meant that most young people would not be able to fulfill the criteria for access to puberty blockers. P7 said:

*Some patients have had to get off puberty blockers because it isn’t possible to get cross-gender hormones before 16 anymore. It’s only in exceptional cases.*/…/ *Then you’re also going to have puberty blockers for 2 years, maximum. If you can start cross-gender hormones by 16 you shouldn’t start [puberty blockers] before 14 years of age either./…/ Not many would benefit from puberty blockers [at 14] because they’ve already gone into puberty.*

This had also led to a situation where “*more people with assigned female gender do not get puberty blockers*” (P2) because their puberty started earlier. However, another perspective highlighted was that people who were assigned male at birth might have a harder time reversing the physical masculinizing changes during puberty, which might affect the possibility of their being perceived in accordance with their gender identity. Other participants explained that the length of the evaluation often meant that it could not be completed in the time available between a care seeker first being seen by the team and the beginning of puberty.

Even though some participants felt that the possibility of getting gender-affirming healthcare had now changed, they perceived the care seekers’ need and desire for gender-affirming medical care as being the same as before the new knowledge support. P7 said: “*I think the expectations are the same.*” The care seekers still hoped to get puberty blockers, hormonal treatment, and mastectomy, although many knew that the possibility had narrowed. Participants such as P5 described cases when adolescents had expressed anger because of the new knowledge support: “*What you can see is that the adolescents who are very convinced about getting hormonal treatment, they are very upset that it has become harder, of course.*” Even the adolescents who fulfilled the criteria of being an exceptional case were worried because “*they feel that their opportunities have shrunk*” (P2).

Since specific gender-affirming care could not be given in many cases, other medical procedures could be provided to help the patient. The new knowledge support attaches great importance to psychosocial support, and this was something provided by all the teams. However, despite the knowledge support, the teams did not seem to have put extra focus on psychosocial support. Some participants explained that they did not have the resources for this, pointing to how the evaluation had become even more extensive. However, P7 said: “*We try referring out a little bit more,*” arguing that the patient might benefit from support from an independent counselor or psychologist who was not involved in the evaluation. P1, who worked with sending referrals to the evaluation teams, said: “*We have no treatment, but supportive counseling … many find it a positive experience to talk to people with trans-specific competence.*” An answer to a question about what was needed for this patient group in the future was “*social support*” (P8). Hormones that were not gender-affirming could be used to stop menstruation and thereby decrease gender dysphoria linked to menstruation. P2 said that they “*recommend progestogen to regulate menstruation.*” Moreover, the new guidelines allowed for an earlier referral to speech and language therapy, which could be helpful for some patients.

### Postponed or denied treatment

The new knowledge support meant that more of the patient group had a longer time before getting treatment. Several participants said that the longer wait had led to an increased level of stress among the care seekers. P2 said: *“Then it means great stress for many adolescents.*” P1 agreed: “*Many [care seekers] are worried.*” The effects for the care seekers were also described as “*frustrating*” and “*painful*”, with the possibility that the young people might isolate themselves while waiting for treatment (P10).

However, some care providers emphasized that waiting was a much larger problem, not solely related to the new guidelines. As the number of care seekers had increased but the available resources had not, waiting times had increased substantially. A combination of long waiting until first contact with the team and the guidelines’ requirement to follow care seekers for at least 2 years meant that in many cases the care seekers would be over 18 years old before treatment was an option. P9 said: “*Most are around 18 or over when they’ve come that far [in the evaluation]/…/ which means that we miss opportunities to help as well.*” The different guidelines for minors and adults could also contribute to the incorrect belief that “*something magic happens around the 18*^*th*^ *birthday*” (P10), meaning that some care seekers believed that if they just turned 18 years old they would receive hormonal treatment, which was not always the case.

Several participants explained that their patients were affected by uncertainty about how long they would have to wait to get treatment and worrying about not being able to get treatment. P2 said:

*And when there is possible treatment, treatment as you see as the light at the end of the tunnel, that you can have this help, and then someone comes and just, it feels like, turns off the light/…/ So, it becomes a huge stress and worry.*

When the new knowledge support was introduced, some 16-year-olds who had thought they would soon start hormonal treatment were told that they had to wait until the age of 18 because of the new guidelines. P4 said that this “*created a little bit of adventure for some of them, with everything from inpatient hospitalization to just more difficult contacts/…/ when the news became really hard for them.*”

The increased amount of time before treatment was seen as positive by some care providers, in some respects. P4 said that the new knowledge support “*makes it easier for me to follow them [the patients] for a longer time.*” This longer time for evaluation was “*a big victory in their own… in their process with themselves*” (P4). The principle of being careful was mentioned. When P6 talked about the evaluation taking a long time, she described this as “*something good*”, since the treatment was about “*very big things*”.

Several participants talked about how the knowledge support could be used as an argument for withholding or postponing treatment, and mentioned the guidelines as clear in these cases and something to lean on. P4 explained:

*It was harder to get … to ask some people to wait before these guidelines. Now, on the other hand, it’s pretty easy, because we can say that these are the rules … so that part has helped us, in a way, in some cases.*

In a similar vein, P10 said that the knowledge support could be something “*to point to*” when telling a patient that hormones were not relevant in their case, reflecting that making this reference might be less damaging to the patient–provider relationship. Longer waiting time was also said be a “*relief*” (P10) for some parents, since treatment after age 18 would no longer be their decision. According to the participants, some parents were “*very, very scared of this, of the treatments and how it will be for their kids*” (P6). It could be hard for parents to accept treatment because of fear and worry. P4 said: “*Many of them are a bit relieved, because it’s often up to 18 now, because then it isn’t a decision that must be made by the guardian.*” P7 said that some parents were “*happy*” because of the new knowledge support.

However, as already mentioned, some care providers wished for more flexibility, and the “*possibility to help more*” (P10). This lack of flexibility could in some cases lead to patients waiting longer for treatment than the healthcare provider thought was necessary. P4 said: “*Some patients have actually been with us since they were children, and this last wait is only a formality.*”

### Opinions and debates affecting care guidelines and care providers

The participants described a range of perceptions of how people outside gender-affirming healthcare influenced not only the knowledge support, but also their work and experiences of it.

### The power of the public debate

According to the participants, there had been a lively public debate on this topic for the past few years. During 2019–2021, Swedish television broadcast documentaries by Uppdrag Granskning on gender-affirming care of children, called the “Trans train”. These were mentioned by several participants. P4 said: *“Before Uppdrag Granskning you didn’t talk about this./…/ Then it came up for discussion”.*

The participants expressed frustration about gender-affirming care being discussed as a political question more than a question about medical healthcare. P4 expressed:

*Still some frustration that it feels like a lot of this work [with new guidelines] has been driven like almost a political question, maybe more than a patient question, because of how Uppdrag Granskning and the public debate have gone the way they have.*

The care seekers were not recognized, as P5 said: “*In some way they get to be the bat in an ideological debate, where you don’t see these individuals on the first hand, instead it’s about politics.*” In connection to this, the participants emphasized that both the debate and the guidelines reflected a simplified picture that failed to account for these being “*very complex issues. But now there’s a lot of pie-throwing and smearing, it’s such a polarized and politicized discussion*” (P9). P9 mentioned several things that are not always considered in the debate or in the production of the guidelines; the hierarchical relationship between healthcare provider and care seeker, issues of patient autonomy and responsibility, and that fact that the evaluation would never be able to be a “fortune teller” and provide 100% certainty in relation to future gender identity.

Several participants thought that the public debate had affected the production and content of the knowledge support. When asked why they thought that the knowledge support had been written in the way that it was, P1 answered: “*I think those kinds of winds of fear based on various political decisions or influences, and so on.*” P9 argued that “*For some reason, there was a very sharp change in the guidelines regarding hormonal treatment, right at the end of the process.*” Conversely, some thought that the content might not have been affected by the public debate. P4 said the debate made the production of the knowledge support go faster: “*I felt that the attention from society speeded up the process, but it was already under investigation before Uppdrag Granskning covered it, for example.*”

### Taxing work and a questioned profession

Every participant mentioned their work as being challenging to various extents. The updated knowledge support made it emotionally hard for some participants, as they could not help the patients in the way they wanted to be helped; that is, through gender-affirming treatment. P3 said: “*And you want to help them as well, and as both an adult and as a healthcare provider, it’s… it’s really hard to make a child disappointed.*” Moreover, working in a questioned area of care was perceived as difficult by some, who felt they needed to take extra precautions to be on the safe side. However, it could also contribute to “*welding together*” the team (P10).

The lack of research on the effects of gender-affirming treatment and the changing characteristics of the patient group were mentioned as a background to the new guidelines and issues that made the job harder. P3 said:

*When the patient and parents have a clear picture of what they need and would make them feel good, and then the healthcare sits on the other side and has very bad… knowledge about/…/ long-time effects, it gets hard for everyone.*

The participants argued that research is needed not only to avoid people being given treatment that they do not need, but also to treat those who are in need. P7 said: “*And that’s for both [situations], so we won’t give the wrong treatment in any way.*”

Some of the participants believed that there is “*a lot of proven experience, a lot*” (P1) which had been ignored in the production of the knowledge support. P2 described the same thing, saying: “*The experience me and my colleagues have built up through seeing these adolescents for many years, it weighed lightly in the end.*” P4 said, in a similar vein: “*Some frustration that we experience, many [providers] in healthcare, that the decision was partly made based on this [opinions of people with little knowledge].*” This was contrasted to how the production of the international guidelines, SOC 8, was characterized by “*listening to people who have a lot of experience working in this field*” (P9).

P1 stated that not all the experts who took part in the production of the knowledge support were listened to: “*[NBHW] chose to listen to/…/ one out of seven, and the others were very critical.*” The members of the expert group involved in producing the knowledge support were reported to be unhappy with the result, as P1 said: “*We were at a conference where several from the expert group who were part of this production [of the knowledge support] were very critical and open with their critique.*” In connection to this, P2 spoke about how the decisions were not made by those working in the field, and mentioned the decision about more restrictive care from Karolinska University Hospital: “*When they took this decision about a stop in Stockholm, then it felt like the ones taking this decision stood far away from the gender-affirming care.*” With regard to the knowledge support, P2 felt that people who did not work in the healthcare of children with gender dysphoria had “*influenced the final result quite a lot.*” P1 thought that the selection of sources used in the SBU report was questionable: “*Unfortunately there is a low trust for these different source references.*” P9 highlighted how the international guidelines were built on other studies due to different “*grading of trustworthiness of the studies*”, but also emphasized that “*there is agreement that more research is needed, and more long-term studies are needed. That’s not under debate.*”

On the other hand, some participants accepted the knowledge support and its sources. When comparing the new knowledge support to the old one, P5 said: “*There is a clearer relation to research.*”

## Discussion

Based on the care providers’ perceptions and experiences, it seems that both before and after the new knowledge support there are differences in healthcare practices across the country, despite the intentions of the new guidelines to provide more uniformity. Some clinics had been more restrictive even before the new knowledge support, and the participants from these clinics argued that it was because they had followed the Dutch Protocol. The new knowledge support refers to the Dutch Protocol several times [1]. The WPATH and SOC 8 also refer to the Dutch Protocol, although they advocate for more liberal guidelines, and it was also the leading research material when the old knowledge support was written. Hence, the Dutch Protocol has been used to argue for both a more liberal and a more restrictive approach, and so it appears to be open to different interpretations, which also seems to be the case with the new Swedish guidelines.

Our results indicate that the different teams have made different interpretations of the new guidelines and the criteria for access. Before the new knowledge support, there were differences between regions. Some participants thought that the new knowledge support provided them with some clarity, but regional differences seemed to remain, and some participants were left with critical thoughts and questions, especially about their limited possibility to provide hormonal treatment. A study from UK showed that parents experienced gender-affirming care services for youth varying depending on the clinicians [26]. Similar to that study, some of our participants expressed a need for a clearer and more unified picture regarding the premises of treatment. A critical question, however, is whether harmonization in itself should be the primary goal; if uniformity comes at the expense of quality at some teams, then greater similarity may not necessarily lead to better care for more care seekers. Regional variation is possibly less problematic than a situation where all teams deliver care of uniformly lower quality, especially as care seekers can seek care at teams that are not closest to them.

Regarding the covariation of gender dysphoria and neuropsychiatric disability, there were very different opinions among participants if such covariation should be an obstacle for accessing care and it seems that the knowledge support had not created a consensus and made the premises clearer. When listing the criteria to consider when deciding on treatment, the knowledge support states that one such criterion is that “The youth has a stable psychosocial situation and no factors that might cloud the certainty of the clinical evaluation (neuropsychiatric or intellectual disability, untreated psychiatric problems including risk of suicide and traumatization, substance usage)” (p. 72); however, it also says that neuropsychiatric disability should not lead to exclusion from diagnosis or treatment when indicated [1]. While some participants were clear that autism and gender dysphoria can exist alongside and that autism should not be a hinder of itself for providing access to care, other participants were more hesitant. A study about ethical and moral challenges in gender-affirming care in Amsterdam, showed that healthcare providers struggled to decide on treatment and diagnosis for young people with autism or an intellectual disability, mentioned the age limit as problematic in some cases where children enter puberty earlier, and challenged the unclear exclusion criteria listed in their national guidelines [27]. A review of clinical guidelines however highlighted that a diagnosis of autism should not, in itself, constitute grounds for exclusion from gender-affirming care [28].

Another issue is the lack of research. The participants expressed a wish for more research, especially regarding the long-term effects of gender-affirming treatment. It is worth noting that the knowledge support allows for the use of puberty blockers and hormonal treatment to be given to youths when participating in research [1]. SBU has called for randomized studies of hormonal treatment for gender dysphoria, but at the same time recognizes this as being non-ethical [3]. Hence, research is argued to be needed to provide treatment for this group, but it is at the same time ethically problematic, which creates an inescapable paradox that needs urgent attention. An extensive review that aimed to identify and collate evidence about the use of puberty blockers and cross-sex hormone therapy among minors diagnosed with gender dysphoria concluded that their literature search did not confirm the commonly held belief that there is limited data on the topic. They identified 277 studies with at least N = 28,056 paediatric GD patients and concluded that the overall evidence supports that gender-affirming medical treatments for paediatric patients with gender dysphoria are both effective (improving mental health, psychosocial well-being, and congruent physical changes) and safe in terms of bone density, cardiovascular, metabolic, or cancer outcomes [29].

The present participants described how the new knowledge support led to longer waiting times for treatment, which was experienced by some as affecting the patients negatively. Waiting for gender-affirming treatment is reported to affect care seeker’s mental health negatively [22,30,31], and it is important for the healthcare system to follow up on how to best support care seekers in this situation. Moreover, resources are needed to decrease the waiting time for starting an evaluation.

Although the knowledge support emphasizes the importance of psychosocial support, the evaluation teams do not seem to have sufficient resources to increase access to such support. Some participants mentioned that referring patients to trans-competent counselors and psychologists outside the evaluation teams could be an advantage. Nevertheless, the participants believed that more psychosocial support is needed, and that appropriate healthcare structures should be developed. However, arguments for increasing psychosocial support must be approached with caution, especially when access to medical treatment remains restricted, to ensure that such support is not misused as a justification for delaying or replacing necessary medical interventions [32].

According to some of the participants, the knowledge support enabled them to avoid giving treatment to patients, which in some cases was seen as positive both by parents and by healthcare providers. This could be problematic, as it does not take into account potentially harmful effects of withholding treatment [33] or the positive effects of treatment that previous studies have shown [12–15,34]. At the same time, the changes within the patient group and the growing number of care seekers have created uncertainty among some healthcare providers, with some questioning the validity of evaluation and gender-affirming care and welcoming a more restrictive approach. Other participants, however, felt constrained by the limited possibilities to provide gender-affirming care, which can be interpreted as the experience not to be trusted in their clinical expertise. This reduced flexibility in clinical assessment also has implications for care seekers. A previous study from the Netherlands showed that standardized protocols and limited flexibility can leave patients feeling marginalized because the evaluation becomes less patient-centered [35]. The authors argued that standardized protocols could allow for more consistent care delivery in theory but in their study, it contributed to longer waiting and could potentially reinforce stereotypical ideals, which are issues that are also brought up in our study. Previous studies and international guidelines have also argued for the importance of adaptable protocols to allow for clinical discretion and to centre the needs of the care seekers [4,36].

The participants felt that in the production of the new knowledge support, proven experience and some kinds of research had been neglected, while voices other than those of the people working with gender-affirming care were perceived to have had an impact. Swedish healthcare should be based on evidence and proven experience [37]. Some of the participating healthcare providers felt that their expertise could not be used in the same way as before, because of the limited room for flexibility when evaluating the need for care. Similar concerns have also been raised in relation to the highly debated and contested Cass review in England, which has been criticized for downplaying the expertise of clinicians and others directly involved in gender-affirming care [38]. The Cass review was published after most of our interviews had been conducted, but it is noteworthy that it cites and commends the Swedish guidelines in several instances [39]. In contrast, the German guidelines, and their assessments of other counties’ guidelines, refer to the Swedish guidelines as deviating from international guidelines and notes that the Swedish guidelines do not bring up methodological limitations in detransition studies, while the Dutch protocol is intensely critiqued and used to argue against puberty blockers and hormones [40].

### Methodological strengths and limitations

The participants in this study had different professions and were recruited from different regions, which enabled us to explore more aspects of the evaluation process and treatment. The open questions in the interview guide allowed the participants to reflect freely, creating extensive material from their discussion of the challenges and facilitators of their work and gender-affirming healthcare for minors in general.

Trustworthiness was improved through using open questions, through discussing the coding, thematization and emergent findings between the authors, and through using many quotations to illustrate the findings. The participants worked in teams and clinics from four of the six Swedish regions that have evaluation teams (and in one additional region), as despite several emails to all teams we did not manage to recruit professionals from the other regions. This means that the findings might not be equally applicable to all teams of Swedish gender-affirming healthcare.

Both authors conducted interviews, coded and analysed the interviews and wrote parts of the paper. Both are trained as medical doctors, but they have different previous experiences of the research topic, different academic experiences, as well as different experiences of gender non-conformity providing both an insider and outsider position in relation to the research topic and facilitating both curious naïve explorations as well as a rigorous knowledge about the research field. No personal relationship existed between the participants and interviewers on beforehand.

## Conclusions and implications

The new knowledge support for treatment of minors with gender dysphoria, published by the Swedish NBHW in December 2022, entails fewer minors receiving puberty blockers and hormonal treatment. This study shows that healthcare providers working with gender-affirming care for minors express differing opinions on the knowledge support. Some are pleased with the new guidelines, and believe the knowledge support is clearer than the old one. Others are more critical, believing that the knowledge support is too rigid and restrictive, and does not take into consideration either clinical experience or all of the relevant research. These different perspective and interpretations did not seem to have emerged with the new knowledge support alone; rather, they seem to reflect long-standing variations in how teams approach gender-affirming care for children and adolescents. Our findings suggest that many reactions to the new guidelines were shaped by pre-existing approaches and ways of working, which influenced care practices both before and after the change. The knowledge support is also perceived to have contributed to care seekers feeling uncertain, stressed and worried. When it comes to “exceptional cases” that should be enabled to access treatment, the criteria are perceived as unclear, which might contribute to geographical differences in care practices. The criteria are also by some experienced as problematic, with, for example, the risk of non-individualized assessment of care need among minors with neuropsychiatric diagnoses. This study might be relevant to healthcare providers working with gender-affirming care. It could also help to inform the production and implementation of future guidelines for the healthcare of minors with gender dysphoria. Finally, the study highlights some restrictions on the potential of care guidelines to create more geographically equitable care; this is an aspect that needs to be further explored.

## Supporting information

S1 File Translated interview guide. (PDF)

S1 Appendix Criteria for treatment of minors with gender dysphoria. (DOCX)
