# Supplementary material for: Healthcare providers’ perceptions of changes in guidelines for care of minors with gender dysphoria in Sweden: An interview study
Source: PLoS One. 2025 Nov 19;20(11):e0336950. doi: 10.1371/journal.pone.0336950 (PMC12629429; doi:10.1371/journal.pone.0336950)
Supplement: S1 File — (PDF) [file pone.0336950.s001.pdf]

Interview guide

The interviews will, in particular, focus on three themes: 1) perception of the effect of the Swedish National Board of Health and Welfare's (Socialstyrelsen) knowledge support (2022) on healthcare; 2) perception of the knowledge supports' impact on the mental health of minors with gender dysphoria, and 3) need for change and improvements. Within all these themes, the interview questions will be adjusted, and follow-up questions will be added to suit the participants' professions and experiences based on what they share.

Demographic questions: gender identity, profession, how long have you been working at your current workplace?

Open-ended initial questions:

1. Can you tell me a bit about your job within this care?

Theme: Perception of the effects of the updated guidelines on healthcare.

1. How did healthcare for minors with gender dysphoria look at your workplace before the guidelines were implemented?
2. How would you describe healthcare for minors with gender dysphoria today?
3. How do you perceive that the updated guidelines from the NBHW (Socialstyrelsen) in 2022 have affected your ability to perform your job?
    a. Have your work tasks changed?
4. How do you perceive that the NBHW's knowledge support has been received by the staff at your workplace?
    a. Why do you think some staff members have a positive perception?
    b. Why do some staff members have a negative/restrictive perception?
5. What do you think was the reason for updating the knowledge support?
    a. What are your thoughts on the possibility that the high prevalence of ADHD and autism among the patient group could have influenced this?
6. How do you perceive the public debate surrounding healthcare before and after the guidelines?

Theme: Consequences of the guidelines for minors with gender dysphoria.

1. How do you feel the updated guidelines have affected the care of young people with gender dysphoria?
    a. In what ways has healthcare improved/deteriorated?
    b. Which specific aspects of care have been affected?
    c. Have fewer resources for hormone treatment meant more resources for another part of the treatment?
2. How do you feel the mental health of young people with gender dysphoria has been affected by the updated knowledge support?
    a. Which part of the guidelines affects mental health?
    b. Patient examples? Remember to de-identify the patient.
3. How do you perceive the desires and opinions of those seeking healthcare?
    a. Any difference compared to before the guidelines were implemented?
4. How do you perceive the desires of the legal guardians regarding healthcare?

    a. Any difference compared to before?
5. How do you perceive the trust of young transgender individuals in healthcare?
    a. Change over time?

Theme: Needs for changes and improvements.

1. What do you think is needed to improve the care of young people with gender dysphoria?

Closing questions:

1. Is there anything you would like to add or something you would like to say that we haven’t touched upon?
2. (stop recording) How did you experience the interview?
