# Supplementary material for: Healthcare providers’ perceptions of changes in guidelines for care of minors with gender dysphoria in Sweden: An interview study
Source: PLoS One. 2025 Nov 19;20(11):e0336950. doi: 10.1371/journal.pone.0336950 (PMC12629429; doi:10.1371/journal.pone.0336950)
Supplement: S1 Appendix — (DOCX) [file pone.0336950.s002.docx]

1. WPATH criteria for providing puberty blockers and hormonal treatment to minors (Coleman et al., 2022):

- Reached Tanner stage 2 of puberty.
- Diagnosed with gender dysphoria.
- Fulfills the criteria for gender incongruence according to ICD-11.
- Gender incongruence is persistent over time.
- Mature enough to take part of the information.
- Mental illness that could interfere with the treatment of gender dysphoria has been evaluated.

1. Swedish NBHW criteria for providing puberty blockers to minors:

- Medical evaluation regarding gender dysphoria has been performed.
- The guardians have given their consent.
- The care-seeker’s psychosocial situation is stable.
- Gender incongruence has been present since childhood.
- Puberty entails suffering.
- Reached Tanner stage 3 of puberty
- Minimum 12 years old.

Swedish NBHW criteria for providing hormonal treatment to minors:

- Medical evaluation regarding gender dysphoria has been performed.
- The guardians have given their consent.
- The care-seeker’s psychosocial situation is stable.
- Gender incongruence has been present since childhood.
- The care-seeker has begun to live socially in accordance with their gender identity.
- The care-seeker is considered to be mature.
- Minimum 16 years old.
